# Synthetic Assembly of Mannose Moieties Using Polymer Chemistry and the Biological Evaluation of Its Interaction towards Concanavalin A

**DOI:** 10.3390/molecules22010157

**Published:** 2017-01-18

**Authors:** Deepti Diwan, Kohei Shinkai, Toshihiro Tetsuka, Bin Cao, Hidenao Arai, Tetsuo Koyama, Ken Hatano, Koji Matsuoka

**Affiliations:** Division of Material Science, Graduate School of Science and Engineering, Saitama University, Sakura, Saitama 338-8570, Japan; deepti992@gmail.com (D.D.); k.shinkai917@gmail.com (K.S.); t.tetsuka.0924@gmail.com (T.T.); bin.cao@colorado.edu (B.C.); harai@mail.saitama-u.ac.jp (H.A.); koyama@fms.saitama-u.ac.jp (T.K.); khatano@fms.saitama-u.ac.jp (K.H.)

**Keywords:** mannose, glycopolymers, concanavalin A, surface plasmon resonance, radical polymerization, kinetic affinity

## Abstract

Protein–carbohydrate interactions exhibit myriad intracellular recognition events, so understanding and investigating their specific interaction with high selectivity and strength are of crucial importance. In order to examine the effect of multivalent binding on the specificity of protein–carbohydrate interactions, we synthesized mannose glycosides as a novel type of glycosylated monomer and glycopolymers of polyacrylamide derivatives with α-mannose (α-Man) by radical polymerization and monitored their strength of interaction with concanavalin A (Con A) by surface plasmon resonance (SPR) detection. In a quantitative test using the Con A-immobilized sensor surface, the kinetic affinity for the synthesized polymers, **8a** (*K_D_* = 3.3 × 10^−6^ M) and **8b** (*K_D_* = 5.3 × 10^−5^ M), were concentration-dependent, showing strong, specific molecular recognition abilities with lectin. Our study showed the enhancement in recognition specificity for multivalent saccharides, which is often mediated by cell surface carbohydrate-binding proteins that exhibit weak affinity and broad specificity for the individual ligands.

## 1. Introduction

Saccharides on cell surfaces are amongst the most complex and prevalent biomolecules that serve a host of biological functions, including physiological as well as pathological functions in the cell via protein–saccharide interactions that facilitate fundamental cell–cell recognition events in processes such as fertilization, development, and the mounting of an immune response [1,2]. Many interactions within the immune system involve specific carbohydrate structures and proteins that recognize and bind them. The processes in which these interactions take place are very diverse and can be part of the infection process or rather can be related to the defense response [3].

The protein–saccharide interactions are usually weak, but are amplified by the multivalent effect of clustered saccharide, the so-called “glyco-cluster effect” [4]. In general, glycans bind at domains defined by shallow pockets on the hydrophilic surface of proteins; hence, affinity regimes of monovalent interactions tend to be rather weak. Multivalency is thus a key concept in lectinology, as it permits not only the enhanced affinity of saccharide-protein interaction but also other phenomena to emerge, such as agglutination [5]. Thus, synthetic glycopolymers with pendent carbohydrate moieties have attracted considerable attention, because these glycopolymers exhibit a large multivalent effect due to densely packed saccharides along the polymer backbone and are applicable as biomaterials and hence a powerful tool in a myriad of biological processes [6,7,8,9] and biomedical applications in biomimetics and human therapeutics [10,11]. Consequently, elucidations of the carbohydrate binding specificity of proteins are of crucial importance in relation to the structure analysis of carbohydrates and the biological roles of sugar chains and proteins [12]. Therefore, in this study, we report the successful synthesis of mannose glycosides as a novel type of glycosylated monomers and its polymer and investigated the binding selectivity of the synthesized substrate with mannose/glucose-binding protein concanavalin A (Con A). We implemented radical polymerization to obtain man-modified copolymers [13,14,15]. In light of the glycoside clustering effect, the strength of the interaction for polymeric substrates was analyzed towards Con A lectin on the basis of a change in the fluorescence of tryptophan at λ_ex_ 295 nm, where the binding showed a decrease in intensity, accompanied by a decrease in UV absorbance with a blue shift and a decrease in fluorescence excited-state lifetimes, probably due to the nature and position of the substituent on the polymeric substrate, thus affecting the association constant as well as changes in fluorescence properties.

Eventually, surface plasmon resonance (SPR) detection was performed to screen the complex formations in real time [16]. In an explorative study, the binding of a synthesized glycopolymer to biosensor-immobilized Con A was examined, and this allowed us to determine both the kinetics parameters and affinity constant *K*_a_ of the interaction, which may further aid in understanding the biological specificity and functions of both the glycoconjugate and the lectin. We anticipate that this synthetic scaffold will offer new means to define the structures of multivalent ligands and densities of binding epitope for specific functions in the lectin–glycan interactions.

## 2. Results and Discussion

**Scheme 1:**
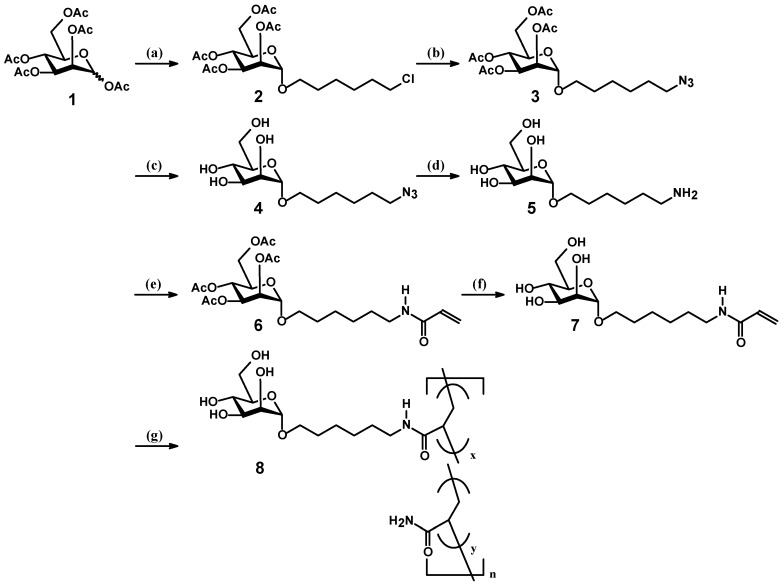
*Reagents and conditions*: (**a**) BF_3_–OEt_2_, 6-Chloro-hexanol, CH_2_Cl_2_, r.t., 18 h; (**b**) *N*,*N*-dimethylformamide (DMF), NaN_3_, 80 °C; (**c**) NaOMe, MeOH, r.t., 2 h; (**d**) triphenyl phosphine (PPh_3_), THF, 0 °C → r.t., 24 h; (**e**) CH_2_=CHCOCl, Et_3_N, MeOH, r.t., 6 h; Ac_2_O, Pyridine, 4 °C → r.t., 15 h; (**f**) NaOMe, MeOH, r.t., 2 h; (**g**) CH_2_=CHCONH_2_, APS, TEMED, water, r.t.

d-(+)-Mannose was selected as the scaffold for the synthesis of the C-1 modified glycopolymer because of its availability and convenient handling. Thus, the copolymers were synthesized by free radical polymerization of de-*O*-acetylated monomer **7** (Scheme 1) [14,17]. Schotten–Baumann reactions were key steps in constructing mannose derivatives with remote terminal acrylamide. The reaction was carried out in an aqueous solution of ammonium persulfate as the initiator and TEMED (*N*,*N*,*N′*,*N*′-Tetramethylethane-1,2-diamine) as the catalyst to provide a water-soluble polymer with erratic tacticity of mannose moieties.

### 2.1. Synthesis of Mannosyl Glycomonomer

Our synthetic scheme is illustrated in Scheme 1. Well-known peracetate is used as a starting material, which is available from a mannose. Thus, the treatment of penta-*O*-acetyl mannopyranose with 6-chlorohexanol in the presence of boron trifluoride diethyl ether (BF_3_–OEt_2_) complex as Lewis acid gave chlorohexyl tetraacetylmannose **2** [18,19], which was further transformed into the desired azidohexyl mannoside **3**. The terminal chlorine atom in the side chain was treated with sodium azide by S_N_2 displacement to afford the corresponding azidohexyl mannoside **3** in almost quantitative yield. Ester exchange reaction of **3** was performed in accordance with Zemplén transesterification, where removal of acetyl protecting groups by treatment with a catalytic amount of sodium methoxide in methanol yielded the desired azido mannoside **4**. The amino group of **5** was condensed with acryloyl chloride by Schotten–Baumann conditions to yield acrylamide derivative **6** in a 56% yield after temporal acetylation (Scheme 1). All ester protections on compound **6** were readily de-*O*-acetylated using Zemplén transesterification conditions to afford **7** [20] in a 93.7% yield.

### 2.2. Polymerization of Mannosyl Glycomonomer

Since monomer synthesis was accomplished, our attention was turned towards the polymer synthesis using the glycomonomer **7**. Radical polymerization of water-soluble *N*-acryloylated derivative **7** with a variation in monomeric feed ratio was carried out in an aqueous solution in order to obtain copolymers containing an anionic group (Scheme 1). Compound **7** was initially polymerized itself in water by means of general polymerization protocol, where the reaction was initiated by ammonium persulfate, following the addition of TEMED as a catalyst [21]. The polymerization reaction proceeded smoothly at room temperature and the viscosity of the reaction mixture gradually increased with progress in the reaction. Two series of molecular weights ($\bar{Mw}$ ~ 178 kDa and 124 kDa) were synthesized for copolymers. In the polymerization reaction, the effects of the mole ratio of monomer/initiator on the polymerization yield and the degree of polymerization of products to ascertain the copolymer composition was estimated from the results of ^1^H-NMR, and the sugar content of the polymer was calculated according to the polymer composition on the basis of formula weight of the glycomonomer unit and the acrylamide unit (Table 1). With the increase in polymerization time, the yield of the polymer increased. However, the molecular weight of the obtained polymers did not evince substantial change. Dialysis against water followed by lyophilization yielded a white powdery glycopolymer **8**. The reaction conditions are summarized in Table 1.

**Figure 1:**
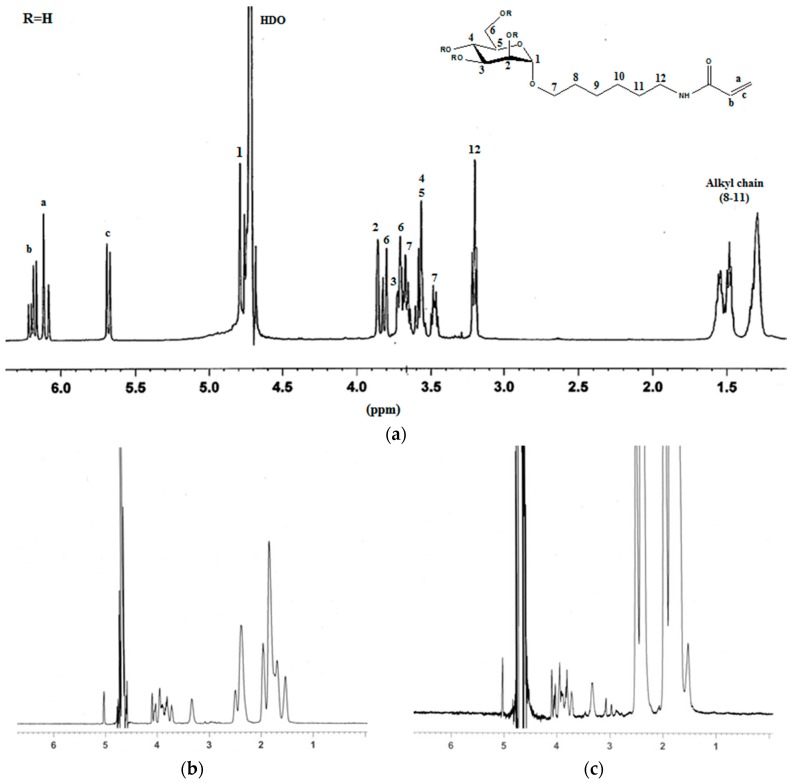
^1^H-NMR of (400 MHz, D_2_O): (**a**) glycomonomer **7**; (**b**) copolymer **8a**; (**c**) copolymer **8b**.

**Figure 2:**
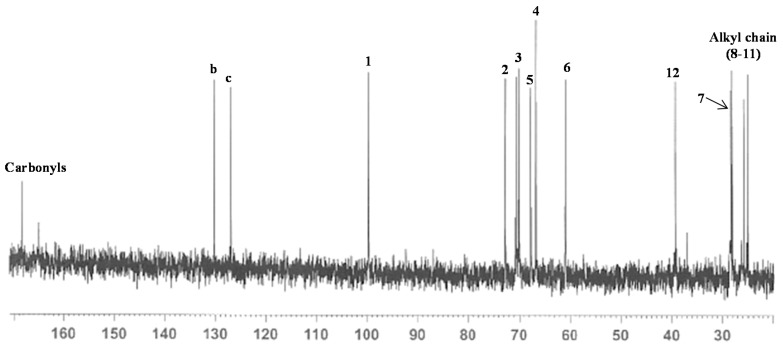
^13^C-NMR spectrum (100 MHz, D_2_O) of glycomonomer **7**.

The chemical structure of the glycomonomer and glycopolymers were elucidated by means of ^1^H-NMR (Figure 1). Protons attached to C=C in the monomer **7** (Figure 1a) at around 5.5–6.5 ppm completely vanished after radical polymerization (Figure 1b) and (Figure 1c), and a broadening of peaks was observed. Moreover, we found the new signal at ca. 1.5–2.5 ppm (Figure 1b,c), and this signal manifests the bond formation between the saccharide moiety and poly-(acrylamide) backbone. The appearance of the requisite number of carbons in the ^13^C-NMR spectrum along with the two characteristic peaks at around 168 ppm assigned to polymer backbone carbonyls further corroborated the assigned structure (Figure 2). The weight-average molecular weight ($\bar{Mw})$ of copolymers were estimated by size exclusion chromatography in a 0.3 M aqueous NaCl solution as the eluent, since we speculate that the addition of an electrolyte (NaCl) facilitates decreased aggregation of the C–C backbone of glycopolymer and hence the hydrophobic interaction, which thus provides the appropriate molecular weight of glycopolymers.

### 2.3. Biological Evaluation of Glycopolymers for Con A

Complete preparation of the glycopolymers with mannose moieties as fishhook was accomplished, and the biological evaluation of the glycopolymers was our next objective. Con A was employed as the model lectin, as it is structurally similar to many animal and bacterial lectins in cell communication events [22,23], besides its property to engage in mono- and multivalent binding, rendering the lectin well suited for investigating the structure/function relationships of both classes. Therefore, to probe into the influence of the nature and densities of sugar residues on the stoichiometry of the cluster, the rate of the cluster formation, the intrinsic affinity and specificity of Con A and the inhibitory potency of the glycopolymers on the specificity of protein–carbohydrate interactions, we synthesized mannose-bearing copolymers with different monomer feed ratios and examined their binding ability towards Con A preliminarily on the basis of fluorescence measurement. Further, the interaction profiles were determined with the aid of SPR, which is known to provide real time binding profiles.

Typical fluorometric measurements of Con A against glycopolymers with mannose moieties were initially carried out. Since no changes of fluorescence spectra upon the addition of a solution of glycopolymer were observed, the trial using the fluorometric technique failed. It was found that a significant change around the tryptophan residue located at or near the binding sites of Con A was not observed. Our attention was turned toward the use of SPR measurement. Thus, in the Con A–glycopolymer interaction study, the lectin was immobilized on the sensor surface of a carboxymethylated dextran-coated (CM5) sensor chip and polymers **8a** and **8b** served as analytes.

**Figure 3:**
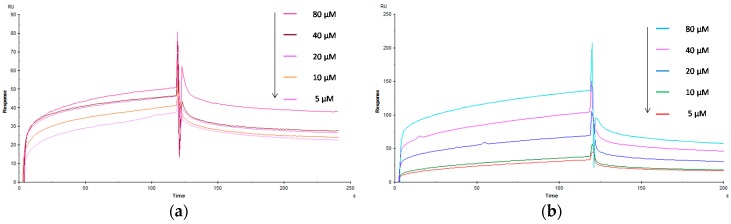
Sensorgram obtained for the binding interactions of Con A with glycopolymers. (**a**) Compound **8a**; (**b**) Compound **8b**. Spike-like signals (inflections) might have been due to the bulk effect [28,29].

Although the Con A-functionalized surface is successfully bound by the glycopolymer, additional binding events can be observed in the sensorgrams. Heterogeneous binding, which is a more effective mimic of relevant biological surfaces, is suggested and allows us to study the ability of the lectin to inhibit multivalent binding modes to a surface. Non-specific binding at the end of a sample injection arising from sugar–sugar interactions is prominently displayed at high concentrations of mannose-modified polymers (Figure 3). This suggests significant sugar–sugar interactions; a pre-organization step between sugars may be a factor in cellular recognition, as opposed to relying solely on the specificity of a protein for a sugar residue [24,25].

However, the kinetic affinities of polymers **8a** (*K_D_* = 3.3 × 10^−6^ M) and **8b** (*K_D_* = 5.3 × 10^−5^ M) to Con A lectin did not appreciably fit to the Langmuir 1:1 binding model, yielding kinetic data that cannot be uniquely determined, thus indicating the heterogeneous nature of the binding, including multivalent binding motifs, which make it difficult to de-convolute individual binding events from the overall system. Interestingly, we observed similar response throughout the dilution series (Figure 3), suggesting the pronounced effect of changes in polymer concentration in the events of Con A binding As the concentration of the glycopolymer is increased, Con A sequestration is enhanced. This is indicative of the polymer’s ability to effectively bind Con A. In addition, dissociation kinetics by **8a** show very slow dissociation (off-rate) relative to **8b**, and such observations support the established hypothesis that the complete dissociation of a multivalent glycopolymers occurs very slowly because all of the individual binding interactions have to dissociate simultaneously from the ligand’s surface [26]. The avidity constant for both the mannose polymers represent a 73-fold enhancement for **8a** and a 4.5-fold enhancement for **8b** compared to its monomer (*K_D_* = 2.4 × 10^−4^ M) [27].

## 3. Experimental Section

### 3.1. General Procedures

All solvents and reagents were available commercially from Kanto Chemical Co., Inc., (Chuo, Tokyo, Japan) and used without further purification. Pyridine (Pyr), Dichloromethane (CH_2_Cl_2_), and *N*,*N*-Dimethylformamide (DMF) were stored over molecular sieves (MS4Å) before use. Tetrahydrofuran (THF) was distilled from the sodium benzophenone ketyl solution, and methanol (MeOH) was stored over MS3Å. Unless otherwise noted, non-aqueous reactions were carried out under a nitrogen and/or argon atmosphere. The optical rotations were determined with a JASCO DIP-1000 digital polarimeter (JASCO Corp., Hachioji, Tokyo, Japan). The ^1^H-NMR and ^13^C-NMR spectra were recorded at 400 MHz on a Bruker AV400 + Cryo spectrometer (Bruker BioSpin Co., Billerica, MA, USA) or at 500 MHz with a Bruker AVANCE 500 spectrometer (Bruker BioSpin Co.) in chloroform-*d* (CDCl_3_) with tetramethylsilane (TMS) or deuterium oxide (D_2_O), including HDO (4.78 ppm) as the internal standard, where chemical shifts are expressed as parts per million (ppm, δ). All reactions were monitored by thin layer chromatography (TLC) on a precoated plate of silica gel 60F_254_ with a thickness of 0.25 mm (E. Merck, Darmstadt, Germany). TLC sheets were prepared for the detection of intermediates and were then sprayed with a solution of 85:10:5 (*v*/*v*/*v*) MeOH-*p*-anisalidehyde concentrated sulfuric acid and heated for a few minutes. Column chromatography was performed on silica gel (Silica Gel 60, spherical neutral, 63–200 μm, E. Merck). By first-order analysis of the spectra, the ring-proton assignments were made in ^1^H-NMR and were supported by the results of HH-cosy or HMQC experiments. Matrix-assisted laser desorption/ionization time-of-flight mass spectra (MALDI-TOF-MS) were obtained using a Bruker Autoflex III spectrometer (Bruker Daltonik GmbH, Bremen, Germany). Elemental analyses were performed with a Fisons EA1108 (Thermo Fisher Scientific, Waltham, MA, USA) on samples extensively dried at 50–60 °C over phosphorous pentoxide for 4–5 h. The weight-average molecular weight ($\bar{Mw}$) of the glycopolymers was estimated by size exclusion chromatography in a 0.3 M NaCl solution using tandem-bonded Shodex SB-803 and SB-804 columns. Calibration curves were obtained using pullulan standards (5.9, 11.8, 22.8, 47.3, 112, 212, 404, and 788 kDa; Shodex P-82). Concanavalin A (Con A; a lectin from jack-bean, *Canavalia ensiformis*) was purchased from J-Oil Mils (# 31209A) and was used without further purification.

### 3.2. Synthesis of Compounds

*6-Cholrohexyl 2,3,4,6-tetra-O-acetyl-α-d-mannopyranoside* (**2**): Boron trifluoride diethyl etherate (BF_3_–OEt_2_) (70.3 mL, 56.0 mmol) was added dropwise over 30 min at 0 °C under an Ar atmosphere to a stirred solution of penta acetate **1** (21.9 g, 56.0 mmol) and 6-chlorohexanol (37.1 mL, 280 mmol) in dichloromethane (50 mL) over molecular sieves (4 Å). The suspension was stirred at room temperature for 18 h. When the TLC of the reaction mixture indicated complete conversion of **1**, the solution was then poured into ice-cold water, followed by an addition of chloroform. The layers were separated, and the aqueous layer was extracted with chloroform and successfully washed with satd aq NaHCO_3_ and brine, dried over anhyd MgSO_4_, filtered, and evaporated in vacuo. Chromatographic purification of the residue using a column of silica gel with 3:1 (*v*/*v*) hexane–ethyl acetate as the eluent yielded pure compound **2** (10.0 g, 46.4%): *R*_f_ 0.88 [1:1 (*v*/*v*) hexane–ethyl acetate]; ^1^H-NMR (400 MHz, CDCl_3_) δ 5.35 (dd, 1H, *J*_3,4_ = 10.0 Hz, H-3), 5.29 (d, 1H, *J*_4,5_ = 9.9 Hz, H-4), 5.23 (m, 1H, H-2), 4.80 (d, 1H, *J*_1,2_ = 1.56 Hz, H-1), 4.28 (dd, 1H, *J*_5,6a_ = 5.3 Hz, *J*_6a,6b_ = 12.2 Hz, H-6a), 4.11 (dd, 1H, *J*_5,6b_ = 2.3 Hz, *J*_6a,6b_ = 12.3 Hz, H-6b), 3.98 (m, 1H, H-5), 3.70 (m, 1H, OCH_a_), 3.55 (t, 2H, *J* = 6.6 Hz, -CH_2_Cl), 3.46 (m, 1H, OCH_b_), 2.16, 2.11, 2.05 and 1.99 (each s, 12H, 4 COCH_3_), 1.79, 1.63, 1.47 and 1.41 (each m, 10H, -OCH_2_(CH_2_)_4_).

*6-Azidohexyl 2,3,4,6-tetra-O-acetyl-α-d-mannopyranoside* (**3**): A suspension of alkyl chloride **2** (10.0 g, 21.4 mmol) and sodium azide (NaN_3_) (6.96 g, 10.7 mmol) in *N*,*N*-dimethylformamide (50 mL) was stirred at 80 °C for 4 h under an Ar atmosphere. When the TLC showed conversion of chloro glycoside into corresponding azide glycoside, the reaction mixture was poured into ice water and extracted with ethyl acetate. The organic layer was successively washed with brine and dried over anhyd MgSO_4_ and the whole mixture was filtered on a pad of Celite to remove insoluble mass and concentrated in vacuo. The crude product was purified by silica gel chromatography using ethyl acetate with a gradient of hexane (1:2 (*v*/*v*)) to afford pure **3** (9.23 g, 98.6%): *R*_f_ 0.53 [1:1 (*v*/*v*) hexane–ethyl acetate]; ^1^H-NMR (400 MHz, CDCl_3_) δ 5.35 (dd, 1H, *J*_2,3_ = 3.4 Hz, *J*_3,4_ = 10.0 Hz, H-3), 5.29 (d, 1H, *J*_3,4_ = 9.88 Hz, H-4), 5.24 (m, 1H, H-2), 4.80 (d, 1H, *J*_1,2_ = 1.56 Hz, H-1), 4.28 (dd, 1H, *J*_5,6a_ = 5.32 Hz, *J*_6a,6b_ = 12.2 Hz, H-6a), 4.11 (dd, 1H, *J*_5,6b_ = 2.44 Hz, *J*_6a,6b_ = 12.2 Hz, H-6b), 3.98 (m, 1H, H-5), 3.69 (m, 1H, OCH_a_), 3.46 (m, 1H, OCH_b_), 3.28 (t, 2H, *J* = 6.8 Hz, -CH_2_N_3_), 2.16, 2.10, 2.05 and 1.99 (each s, 12H, 4 COCH_3_), 1.62 (m, 8H, -OCH_2_(CH_2_)_4_); ^13^C-NMR δ (100 MHz, CDCl_3_) 170.63, 170.11, 169.91, 169.74 (C=O), 97.58 (C-1), 69.71 (C-2), 69.12 (C-3), 68.46 (C-5) 68.29 (OCH_2_), 66.29 (C-4), 62.55 (C-6), 51.36 (CH_2_N_3_), 29.12, 28.74, 26.47 and 25.72 (CH_2_), 20.90, 20.73 and 20.69 (CH_3_).

*6-Azidohexyl α-d-mannopyranoside* (**4**): Sodium methoxide (NaOCH_3_) (283 mg, 5.24 mmol) was added to a solution of acetate **3** (6.20 g, 13.1 mmol) in MeOH (60 mL) at room temperature under N_2_ atmosphere, and the reaction mixture was stirred for 30 min. When complete disappearance of acetate **3** on TLC was observed, IR-120B (H^+^) was added (the pH of the solution was adjusted to range from pH 3 to 5). After stirring the mixture for 15 min, the resin was filtered off and washed successively with methanol and the solvent was removed in vacuo to yield the corresponding alcohol **4** (3.94 g, 98.5%) as a light-yellow syrup, which was used for the next step without further purification: *R*_f_ 0.26 [7:1 (*v*/*v*) chloroform–methanol]; 1H-NMR (400 MHz, CDCl_3_) δ 4.81 (br s, H-1), 3.97 (br s, 1H, H-2), 3.93 (br, 1H, H-6a), 3.82 (dd, 1H, *J*_2,3_ = 2.76 Hz, *J*_3,4_ = 9.56 Hz, H-4), 3.77 (m, 1H, H-6b), 3.64 (m, 1H, OCH_a_), 3.50 (m, 1H, H-3), 3.45 (m, 1H, H-5), 3.39 (m, 1H, OCH_b_), 3.27 (t, 2H, *J* = 6.8 Hz, -CH_2_N_3_), 1.50 (br, 8H, -OCH_2_(CH_2_)_4_); ^13^C-NMR δ (100 MHz, CDCl_3_), 100.04 (C-1), 72.20 (C-5), 71.68 (C-3), 71.06 (C-2), 67.69 (OCH_2_), 66.18 (C-4), 60.97 (C-6), 51.36 (CH_2_N_3_), 29.24, 28.75, 26.51 and 25.71 (CH_2_).

*6-Aminohexyl α-d-mannopyranoside* (**5**): To a stirred solution of **4** (3.94 g, 12.9 mmol) and water (6.50 mL), tetrahydrofuran (17.5 mL) was added at room temperature under a N_2_ atmosphere, and the mixture was cooled to 0 °C for 0.5 h. Triphenylphosphine (TPP) (3.75 g, 15.7 mmol) was then added to the mixture at 0 °C. The mixture was allowed to warm to room temperature and was stirred overnight. When the TLC evinced the complete transformation of azide alcohol **4** into the corresponding amine **5** in chloroform–methanol–water (6.5:2.5:0.4 (*v*/*v*/*v*)), the reaction mixture was then poured into ice water and washed with chloroform to remove triphenylphosphine oxide, and the obtained aqueous layer was then lyophilized to obtain **5** (quantitative) as a white mass. ^1^H-NMR (500 MHz, D_2_O) δ 4.80 (d, 1H, *J*_1,2_ = 1.55, H-1), 3.87 (dd, 1H, *J*_1,2_ = 1.7 Hz, *J*_2,3_ = 3.4 Hz, H-2), 3.82 (m, 1H, H-6a), 3.73 (m, 1H, H-4), 3.71 (m, 1H, H-6b), 3.67 (m, 1H, OCH_a_), 3.61 (s, 1H, H-3), 3.57 (d, 1H, *J*_5,6a_ = 1.45 Hz, H-5), 3.49 (m, 1H, OCH_b_), 2.63 (t, 1H, *J* = 7.18 Hz, -CH_2_NH_2_), 1.44 (m, 8H, -OCH_2_(CH_2_)_4_); MALDI-TOF-MS Calcd for [M]^+^ 279.33, [M + H]^+^ 280.33. Found: *m*/*z* 279.978, [M + Na]^+^ 302.33. Found: *m*/*z* 301.982, [M + K]^+^ 318.33. Found: *m*/*z* 317.957.

*6-Acrylamidohexyl 2,3,4,6-Tetra-O-acetyl-α-d-mannopyranoside* (**6**): To a solution of amine **5** (300 mg, 1.07 mmol) and dry MeOH (3.0 mL), triethylamine (0.92 mL, 6.60 mmol) was added, followed by a dropwise addition of acryloyl chloride (220 μL, 2.72 mmol) at 0 °C under an Ar atmosphere, and the reaction mixture was stirred for 30 min at the same temperature and was then continuously stirred at room temperature. When the TLC showed conversion of amine **5** to the corresponding acrylamide, the solution was then concentrated in vacuo. *R*_f_ 0.80 [6.5:2.5:0.4 (*v*/*v*/*v*) chloroform–methanol–water]. An acetylation of acrylamide was further performed in order to purify the products. The residue was allowed to react with Ac_2_O (5.0 mL, 52.8 mmol) in pyridine (5.0 mL) at 0 °C under an Ar atmosphere, and the reaction mixture was stirred overnight at room temperature. The mixture was diluted with CHCl_3_ and then poured into ice-cold water. The mixture was partitioned, and the organic layer was successively washed with 1 M aq H_2_SO_4_, sat aq NaHCO_3_, and brine, dried over anhyd MgSO_4_, filtered, and concentrated in vacuo. Chromatographic purification of the residue on silica gel with 1:3 (*v*/*v*) *n-*hexane–ethyl acetate as the eluent to yield pure **6** (0.70 g, 56%): *R*_f_ 0.63 [ethyl acetate]; ${\left[\propto \right]}_{D}^{34}$ + 38.2° (*c* 1.00, CHCl_3_); ^1^H-NMR (400 MHz, CDCl_3_) δ 6.28 (dd, 1H, *J*_gem_ = 1.5 Hz, *J*_trans_ = 17.0 Hz, -CH=CH_2_), 6.09 (dd, 1H, *J*_cis_ = 10.3 Hz, *J*_trans_ = 17.0 Hz, -CH=CH_2_), 5.71 (1H, amide NH), 5.62 (dd, 1H, *J*_gem_ = 1.5 Hz, *J*_cis_ = 10.1 Hz, -CH=CH_2_), 5.34 (dd, 1H, *J*_2,3_ = 3.3 Hz, *J*_3,4_ = 10.0 Hz, H-3), 5.28 (t, 1H, *J*_3,4_ = *J*_4,5_ = 9.9 Hz, H-4), 5.22 (dd, 1H, *J*_1,2_ = 1.74 Hz, *J*_2,3_ = 3.3 Hz, H-2), 4.80 (d, 1H, *J*_1,2_ = 1.6 Hz, H-1), 4.28 (dd, 1H, *J*_5,6a_ = 5.3 Hz, *J*_6a,6b_ = 12.2 Hz, H-6a), 4.11 (dd, 1H, *J*_5,6b_ = 2.5 Hz, *J*_6a,6b_ = 12.2 Hz, H-6b), 3.98 (m, 1H, *J*_4,5_ = 9.6 Hz, *J*_5,6a_ = 5.3 Hz, *J*_5,6b_ = 2.5 Hz, H-5), 3.69 (m, 1H, *J*_gem_ = 9.7 Hz, *J*_vic_ = 6.4 Hz, OCH_a_), 3.45 (m, 1H, *J*_gem_ = 9.7 Hz, *J*_vic_ = 6.3 Hz, OCH_b_), 3.34 (m, 2H, -CH_2_NH), 2.16, 2.10, 2.05 and 1.99 (each s, 12H, 4 COCH_3_), 1.48 (m, 8H, -OCH_2_(CH_2_)_4_); ^13^C-NMR δ (100 MHz, CDCl_3_), 170.69, 170.16, 170.06 and 169.75 (C=O), 165.55 (NHCO), 130.97 and 126.15 (-CH= CH_2_), 97.53 (C-1), 69.71 (C-2), 69.20 (C-3), 68.46 (C-5), 66.27 (C-4), 62.57 (C-6), 39.52 (NHCH_2_), 29.48, 29.02, 26.69 and 25.99 (CH_2_), 20.90, 20.75 and 20.71 (CH_3_); MALDI-TOF-MS Calcd. for [M]^+^ 501.52, [M + H]^+^ 502.52. Found: *m*/*z* 502.204, [M + Na]^+^ 524.52. Found: *m*/*z* 524.203, [M + K]^+^ 540.52. Found: *m*/*z* 540.185. Anal. Calcd for C_23_H_35_NO_11_·1.2 H_2_O: C, 55.081; H, 7.034; N, 2.793; O, 35.092. Found: C, 52.805; H, 7.206; N, 2.677.

*6-Acrylamidohexyl α-d-mannopyranoside* (**7**): To a stirred solution of acetate **6** (200 mg, 399 μmol) in methanol (10 mL), sodium methoxide (8.64 mg, 1.60 mmol) was added at room temperature under a N_2_ atmosphere, and the solution was further stirred for 1 h. After the reaction, IR-120B (H^+^) resin was added to adjust the pH of the solution to a range from pH 3 to 5, and the suspension was filtered. The filtrate was concentrated in vacuo to yield pure acrylamide **7** (125 mg, 93.7%): *R*_f_ 0.06 [5:4:1 (*v*/*v*/*v*) chloroform–ethyl acetate–methanol]; ^1^H-NMR (500 MHz, D_2_O) δ 6.55 (dd, 1H, *J*_gem_ = 10.2 Hz, *J*_trans_ = 17.1 Hz, -CH=CH_2_), 6.09 (d, 1H, *J*_trans_ = 17.0 Hz, -CH=CH_2_), 5.68 (d, 1H, *J*_cis_ = 10.2 Hz, -CH=CH_2_), 4.77 (br s, H-1), 3.86 (m, 1H, H-2), 3.81 (d, *J*_5,6a_ = 12.1 Hz, H-6a), 3.73 (m, 1H, H-3), 3.70 (m, 1H, *J*_6a,6b_ = 5.15 Hz, H-6b), 3.66 (m, 1H, OCH_a_), 3.59 (m, 1H, H-4, H-5), 3.48 (m, 1H, OCH_b_), 3.20 (t, 2H, *J* = 6.85 Hz, -CH_2_NH), 1.53 (m, 8H, -OCH_2_(CH_2_)_4_); ^13^C-NMR δ (100 MHz, D_2_O), 168.44 (NHCO), 130.09 and 126.91 (-CH= CH_2_), 99.65 (C-1), 72.70 (C-2), 70.36 (C-3), 67.77 (C-5), 66.75 (C-4), 60.90 (C-6), 39.33 (NHCH_2_), 28.35, 28.11, 25.78 and 25.01 (CH_2_).

*Radical polymerization* (**8**): Copolymerization: A solution of carbohydrate monomer **7** (30 mg, 100 μmol) in deionized water (0.3 mL) was deaerated under reduced pressure for a few minutes, and TEMED (3 μL, 20 μmol) and APS (2.3 mg, 10 μmol) were then added. The mixture was stirred at room temperature for 24 h. Polymerization was terminated by a 1 M aqueous pyridine-acetic acid buffer, pH 5.6 (1 mL). The viscous solution was dialyzed against distilled water, followed by direct lyophilization to yield a corresponding white powdery glycopolymer **8a** or **b**. The results of radical polymerization are summarized in Table 1: **8a** (71%): $\bar{Mn}$ 189 kDa, $\bar{Mw}\;$214 kDa, $\bar{Mn}/\bar{Mw}$ 1.1; **8b** (90%): $\bar{Mn}$ 116 kDa, $\bar{Mw}\;$124 kDa, $\bar{Mn}/\bar{Mw}\;$ 1.0.

### 3.3. Evaluation of the Interaction Kinetics of Glycopolymers for Lectin

We examined the affinity interaction between the glycopolymers and Con A lectin by employing spectrofluorimetric and surface plasmon resonance (SPR) detection methods. Fluorescence emission and excitation (a slit width of 5.0 nm with a medium scanning speed) spectra measurement was carried out at ambient temperature in a Teflon-stoppered cuvette (12.5 mm wide × 45 mm high) containing a 3.0 mL sample. The interval of sampling was 1 nm. Emission spectra of Con A induced by excitation at 295 nm and were recorded with a Shimadzu RF-5300PC fluorescence spectrophotometer. The cuvette was mounted in a thermostated holder, and the measurement was carried out at 4 °C. The concentration of Con A was estimated to be 0.78 μM by using an absorption coefficient at 280 nm ($E_{280}^{0.1\%}=1.20$ in 50 mM Hepes buffer, 0.15 M NaCl, 0.005 M KCl, pH 7.5).

Saccharide–protein interactions analyzed according to SPR measurements were obtained with a BIACORE X100 (GE Healthcare Uppsala, Sweden) by Con A lectin immobilization on gold substrates of carboxymethylated dextran-coated (CM5) sensor chips [30] by an amine-coupling procedure, using a 0.01 M HEPES (4-(2-hydroxyethyl)-1-piperazineethanesulfonic acid) buffer containing 0.15 M NaCl, 0.03 M EDTA, and 0.50% (*v*/*v*) Surfactant P20 (pH 7.4) as an eluent and a flow rate of 10 μL/min. The stock solution of Con A with a 1 mg/mL concentration was prepared in deionized water. The chip surface was activated by derivatization with a freshly prepared mixture of an aqueous solution of 0.4 M 1-ethyl-3-(3-dimethylaminopropyl) carbodiimide (EDC) and 0.1 M *N*-hydroxysuccinimide (NHS) (1:1, *v*/*v*). The mixture was injected into the chip flow channels sequentially at a flow rate of 10 μL/min, which was then followed by injection of lectin solution at a 0.01 mg/mL concentration in a 0.01 M sodium acetate buffer (pH 4.5). Finally, the unoccupied sites were blocked by injection of 1 M ethanolamine-HCl (pH 8.5) for 7 min at a rate of 10 μL/min. Each step was followed by buffer rinses for a few minutes. Both activation and immobilization procedure were performed at 25 °C.

To calculate the kinetics of the interaction of polymers **8a** and **8b**, separate experiments were performed on a flow cell containing 1000 and 400 RU of dimeric Con A (active surface), respectively. A concentration series of polymers **8a** and **8b** were injected onto the Con A surface (Table 2). Between binding cycles, the Con A surface was regenerated with a 30 s pulse of the 0.01 M glycine-HCl (pH 1.5). The working concentration of the polymers were prepared by diluting their stock solutions in the running buffer containing a 0.01 M HEPES buffer, 0.15 M NaCl, 1 mM Ca^2+^ (or Mn^2+^), and 0.05% (*v*/*v*) Surfactant P20 (pH 7.4).

The gold surface used was functionalized with a rather high percentage of amine-terminated dextran that was subsequently coupled to a tethered Con A. This places the dimeric Con A close enough to undergo bivalent binding with the mannoside polymer, preventing an accurate determination of monovalent association and dissociation rate constants but allowing equilibrium constants of highly multivalent systems to be determined. To study Con A binding to the corresponding polysaccharides **8a** and **8b**, the sensorgrams measured at several concentrations were analyzed with the Langmuir binding (1:1) using BIAevaluation software (GE Healthcare Uppsala, Sweden). The affinity profile of polymers **8a** and **8b** for Con A is shown in Figure 3. The *K_D_* value of polymers **8a** and **8b** to Con A were 3.3 × 10^−6^ M and 5.3 × 10^−5^ M, respectively.

## 4. Conclusions

In summary, we report the successful synthesis of a novel type of glycosylated monomer and its polymers, compounds **8a** and **8b**. Synthesized glycopolymers were used as analytes in SPR studies involving Con A-immobilized surfaces, and binding affinity experiments were strategically carried out at physiological pH. We observed that **8a** bound to Con A with a 15-fold increase in affinity (*K_D_*) compared to **8b**. The trend in such results agree with previous reports in terms of a steric effect in that a higher number of ligand-conjugates do not always represent any real advantage for carbohydrate–protein interactions [31]. Therefore, mannose residues seem to be the main factor in enhancing multiple interactions for the binding stoichiometry, the rate of binding, the potency, and the stability of Con A clustering. Therefore, this work is a crucial example to demonstrate how the well-defined glycopolymers with various pendant tops have a critical influence on the lectin–multivalent interaction and the stability of the Con A–glycopolymer cluster regarding the change in complex formation parameters.

## Figures and Tables

**Table 1:** Polymerizations of carbohydrate monomer with acrylamide.

Compound	Monomer Ratio ^a^	Total Yield ^b^ (%)	Polymer Composition ^c^			Sugar Content (wt %)	$\bar{Mw}$ ^d^ (kDa)	$\bar{Mw}/\bar{Mn}$
**8**			**x**	**y**	**n**			
**a**	1:10	71	1	12	532	23.8	178	1.1
**b**	1:20	90	1	130	310	3.6	124	1.0

^a^ Carbohydrate monomer/acrylamide. ^b^ Total yields were calculated on the basis of quantities of monomers used. ^c^ Polymer compositions of sugar unit/acrylamide unit were estimated on the basis of the results of ^1^H-NMR. ^d^ The weight-average molecular weights were estimated by size-exclusion chromatography in 0.3 M aq NaCl solution using tandem-bonded Shodex SB-803 and SB-804 columns.

**Table 2:** Kinetic parameters for Con A–glycopolymer interactions on the Con A surface.

Compound	Working Concentrations (μM)					Flow Rate (μL/min)	Contact Time (min)	Dissociation Time (min)
**8a**	5	10	20	40	80	30	1	1
**8b**	5	10	20	60	80	30	2	10
